# Use of N-Acetylcysteine in the Management of Isoniazid-Induced Liver Injury in a Tuberculosis Patient: A Case Report

**DOI:** 10.7759/cureus.74445

**Published:** 2024-11-25

**Authors:** Armand Ntchana, Robert Muhumza

**Affiliations:** 1 Family Medicine, Rapides Regional Medical Center, Alexandria, USA; 2 Gastroenterology and Hepatology, Rapides Regional Medical Center, Alexandria, USA

**Keywords:** drug-induced liver injury (dili), hepatotoxicity, isoniazid, n-acetylcysteine (nac), tuberculosis treatment

## Abstract

Drug-induced liver injury (DILI) is a rare but significant cause of acute liver failure, often challenging to diagnose due to its clinical similarity to other liver conditions. Since most drugs are metabolized by liver enzymes, the liver is at risk for hepatotoxicity. Although DILI has a low incidence in clinical practice, it remains a critical consideration for patients on potentially hepatotoxic medications. Acetaminophen is the most commonly implicated drug in DILI cases and is prioritized in toxicology screenings. Effective management of DILI requires the prompt discontinuation of the offending drug and supportive care. This case report discusses a 65-year-old male patient who developed elevated liver enzymes three weeks after starting tuberculosis treatment, raising suspicion of DILI. This report explores the diagnostic process, management strategies, and therapeutic role of N-acetylcysteine (NAC), emphasizing its mechanism of action, current clinical applications, and potential future uses in treating DILI.

## Introduction

Drug-induced liver injury (DILI) is a rare but significant cause of acute liver injury, often challenging to diagnose due to its clinical similarity to other liver conditions [1]. Since most drugs are metabolized by liver enzymes, the liver is at risk for hepatotoxicity. Although DILI has a low incidence in clinical practice, it remains a critical consideration for patients on potentially hepatotoxic medications. Acetaminophen is the most commonly implicated drug in DILI cases and is prioritized in toxicology screenings. Effective management of DILI requires the prompt discontinuation of the offending drug and supportive care. However, it is crucial to remember that each DILI case is unique, and individualized patient management is essential. This case report discusses a 65-year-old male patient who developed elevated liver enzymes three weeks after starting tuberculosis (TB) treatment, raising suspicion of DILI. The report explores the diagnostic process, management strategies, and therapeutic role of N-acetylcysteine (NAC), emphasizing its mechanism of action, current clinical applications, and potential future uses in treating DILI.

Adverse drug reactions, including DILI, are relatively rare but can have severe consequences when they occur. Since most drugs are metabolized by liver enzymes, hepatotoxicity remains a critical risk, particularly with the use of hepatotoxic drugs and other xenobiotics. DILI is associated with significant liver-related morbidity and mortality, especially in cases where the causative drug is not promptly identified and discontinued. The three primary causes of DILI are acetaminophen (Tylenol)-induced DILI, idiosyncratic DILI, and DILI of an indeterminate cause [1]. Acetaminophen is the most frequent cause of acute liver injury, accounting for approximately 47% of reported cases. In addition to acetaminophen, anti-TB medications, including rifampin, isoniazid, and pyrazinamide, as well as antiepileptic drugs like phenytoin, are known to cause idiosyncratic DILI [1].

Several factors can predispose individuals to DILI, including advanced age, alterations in the gut microbiome, pre-existing liver conditions, diabetes, and other comorbidities such as human immunodeficiency virus (HIV) [2]. Distinguishing DILI from other liver conditions is particularly challenging, as it can mimic clinical presentations seen in viral hepatitis, autoimmune hepatitis, alcoholic liver disease, and both acute and chronic liver failures. Symptoms of DILI, including jaundice, pruritus, dark stools and urine, ascites, and abnormal liver function, often overlap significantly with those of other liver disorders [2]. Laboratory tests usually show elevated liver enzymes, which can be either asymptomatic or symptomatic [3]. Depending on the specific enzyme profile, these elevated enzymes may indicate hepatocellular injury or hepatobiliary involvement.

The management of DILI involves the prompt cessation of the offending drug and supportive care. Additional approaches include hydration, continuous monitoring of laboratory values, coagulation studies, and liver function tests [4]. In cases where liver function is severely impaired, coagulopathy may develop, necessitating the administration of vitamin K. NAC is another recommended therapy for DILI management due to its antioxidant and hepatoprotective properties.

This paper presents a case report of a 65-year-old male patient who was referred to the emergency department after elevated liver enzymes were detected at his primary care facility. The patient was undergoing treatment for TB and had a history of social alcohol consumption but without evidence of excessive alcohol use or other organ dysfunctions. Imaging studies and liver chemistries supported the diagnosis of DILI. This case explores the clinical presentation, diagnostic workup, and therapeutic interventions, with a focus on the role of NAC in managing DILI.

## Case presentation

**Figure 1:**
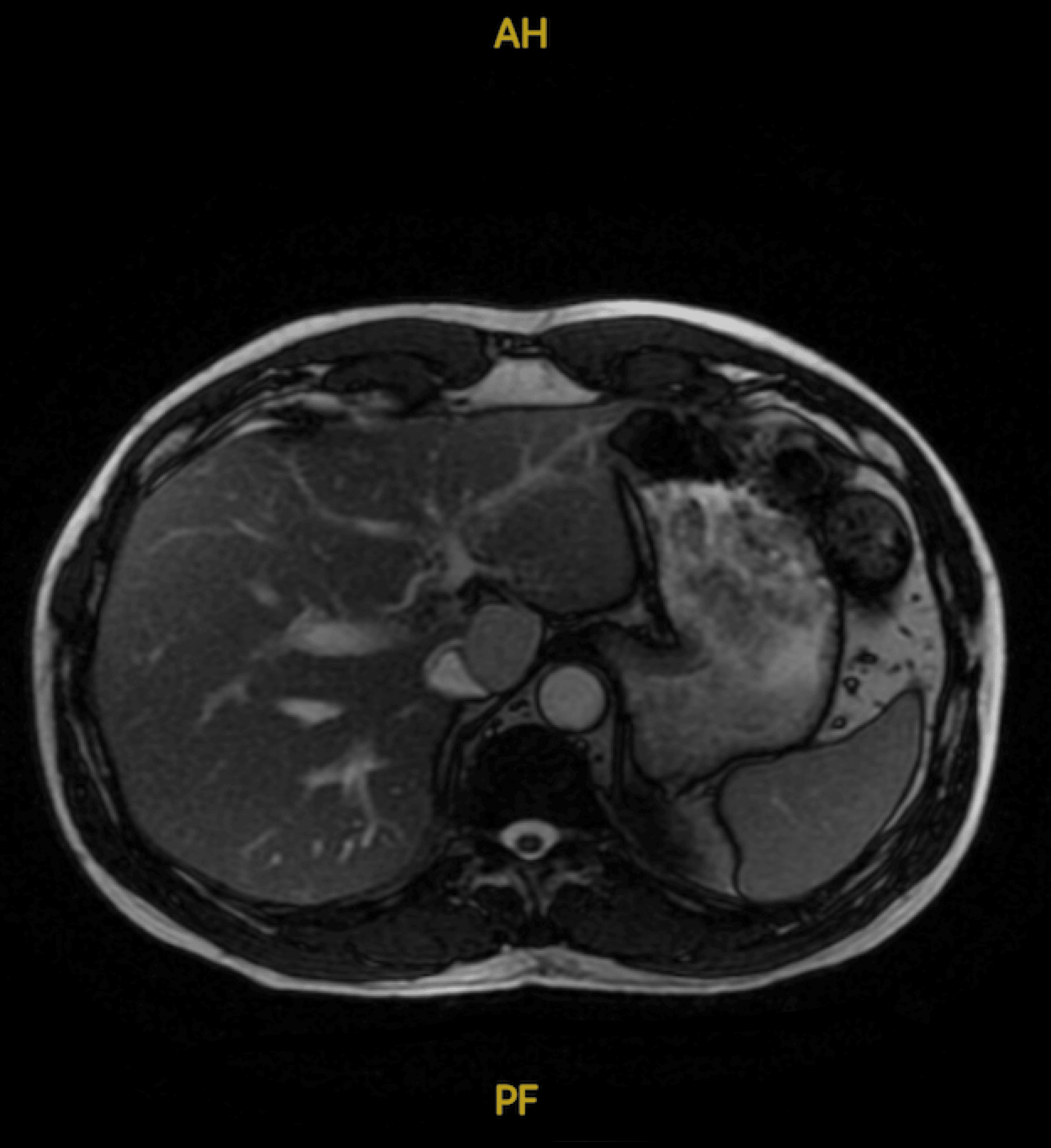
Liver MRI: smooth, regular appearance, with uniform signal intensity, normal vascular structures, and no signs of abnormalities, such as lesions, fatty infiltration, or biliary obstruction. Gallbladder wall: 2-3 mm.

A 65-year-old male patient was referred from a prison hospital to the emergency department with a primary complaint of elevated liver enzymes. Additional symptoms included nausea and jaundice. The patient had no significant past medical history and was not taking any medications. He had been recently diagnosed with TB and initiated on an eight-week course of first-line anti-TB therapy, consisting of isoniazid, ethambutol, pyrazinamide, and rifampicin. After two weeks of treatment, routine monitoring revealed a significant elevation in liver enzymes, exceeding 50 times the normal range. This prompted the immediate discontinuation of therapy and hospital admission for further evaluation. The patient reported social alcohol consumption, typically two to four drinks on weekends, with the last drink consumed six months before his incarceration. He denied any chronic conditions, recent acute illnesses, or trauma. An abdominal ultrasound and computed tomography (CT) scan were performed, both of which were unremarkable. No lesions, obstruction, or other abnormalities were observed in the liver or cystic ducts (Figure 1).

Upon admission, significant laboratory findings were observed.

The Model for End-Stage Liver Disease (MELD) score is a numerical scale used to assess the severity of chronic liver disease and predict mortality risk in patients awaiting liver transplantation. It is calculated by incorporating three laboratory values: serum bilirubin, serum creatinine, and international normalized ratio (INR) for prothrombin time (PT). The score ranges from 6 to 40, with higher scores indicating more severe liver dysfunction and a greater likelihood of mortality. The MELD score is crucial for prioritizing patients on transplant waiting lists, ensuring that those most in need receive timely medical intervention. The patient's calculated MELD score was calculated at 18. The initial impression included elevated transaminases, TB, and severe liver injury, likely due to drug toxicity. The diagnosis of transaminitis was based on significantly elevated liver enzymes, with aspartate aminotransferase (AST) and alanine aminotransferase (ALT) levels exceeding three times the upper limit of normal, a total bilirubin (T.Bili) level of 7.2 mg/dL, and elevated lipase levels of 165. A magnetic resonance cholangiopancreatography (MRCP) was performed to rule out any secondary causes of pancreatic injury and to confirm the patency of the bile ducts. The TB diagnosis was supported by the patient’s recent history of positive TB testing and ongoing treatment. Severe liver injury secondary to DILI was considered after ruling out other potential causes, such as viral hepatitis, based on the negative hepatitis panel.

Continuous laboratory monitoring and gastrointestinal consultation were initiated. On day two, laboratory values showed a further increase in T.Bili from 7.2 to 7.4 mg/dL, with a direct bilirubin of 6.5 mg/dL and an indirect bilirubin of 0.9 mg/dL. AST and ALT remained elevated at 1912 and 2267 units/L, respectively. Iron ferritin levels also increased. The coagulation profile showed a normal PT and INR. In this case, the absence of encephalopathy and a normal PT/INR suggested that acute liver failure was unlikely, as it is typically characterized by the rapid deterioration of liver function, coagulopathy, and altered mental status. Urinalysis results revealed elevated urobilinogen levels (2.0 Eu/dL) and increased urine red blood cells (RBCs). The impression on day two remained severe DILI, with isoniazid being the most likely culprit. 

The Liver Toxicity Score (LTS) is a system used to assess the risk of liver injury associated with various medications, including those used to treat TB. The score is calculated based on factors such as the incidence of hepatotoxicity in clinical studies, the severity of liver enzyme elevations, and the likelihood of dose-related toxicity. Each medication is assigned a score, typically ranging from 0 to 3, with higher scores indicating a greater risk of liver damage. Among TB medications, isoniazid has the highest LTS, often rated at 2-3, due to its propensity to cause hepatotoxicity, especially in patients with risk factors such as advanced age or pre-existing liver disease [2].

On day three, a gastrointestinal consult suggested the diagnosis of isoniazid-induced hepatitis, attributing the elevated ferritin levels to acute liver injury. Recommendations from the gastrointestinal physician included close monitoring of liver enzymes, withholding anti-TB medications, and continuing intravenous (IV) hydration. This approach was supported by an infectious disease consult, who agreed to withhold anti-TB medications due to elevated liver enzyme levels.

By day six, the infectious disease team recommended reintroducing anti-TB medications one at a time, except for isoniazid (INH), once liver enzymes stabilized. However, persistent hyperbilirubinemia contradicted this plan. On day seven, laboratory results showed worsening liver function, with a T.Bili of 9.3 mg/dL, AST of 1194 units/L, and ALT of 2174 units/L. The PT was 11.4 seconds, and the INR remained at 1.1. By day eight, T.Bili had further increased to 10 mg/dL, while PT/INR remained stable. A bedside consultation by the gastrointestinal and infectious disease teams recommended initiating NAC therapy, as the MELD score remained at 18.

NAC is primarily recognized as an antidote for acetaminophen overdose, with an initial loading dose of 140 mg/kg administered intravenously, followed by a maintenance dose of 70 mg/kg every four hours for a total of 17 doses (or 72 hours of treatment). For cases of acute liver injury outside of acetaminophen toxicity, although there is no standardized dosing protocol, the American Association for the Study of Liver Diseases (AASLD) suggests utilizing a regimen similar to that used for acetaminophen overdose. This involves administering a loading dose of 150 mg/kg IV over one hour, followed by 50 mg/kg IV over four hours, and then 100 mg/kg IV over the subsequent 16 hours. This approach is based on NAC's antioxidant properties and its potential benefits in mitigating liver injury.

On day nine, after the second dose of NAC, a slight improvement in liver biochemistry was observed, with a reduction in T.Bili to 9.2 mg/dL, AST to 1088 units/L, and ALT to 2039 units/L. From day 10 onward, there was a significant decline in liver enzyme levels. By day 12, following the sixth dose of NAC, the patient's T.Bili had decreased to 5.9 mg/dL, AST to 644 units/L, and ALT to 1377 units/L. The INR remained low at 1.0, and the PT was 10.8 seconds. The MELD score improved to 15, and daily monitoring of laboratory values and the MELD score was recommended.

On day 13, liver enzyme levels continued to improve, with AST at 475 units/L, ALT at 1131 units/L, and T.Bili at 4.5 mg/dL. However, the INR remained low at 1.0, and the PT was 10.5 seconds (Table 1). The patient was discharged on day 17 and returned to the prison facility for continued monitoring of liver enzyme levels. Recommendations were made for follow-up with a pulmonologist and a gastroenterologist before gradually reinitiating anti-TB medications or considering an alternative regimen based on the patient's response. 

**Table 1:** Aspartate aminotransferase (AST), alanine aminotransferase (ALT), total bilirubin (T.Bili), and prothrombin time (PT) levels over 17 days, indicating a gradual decline across the period.

Day	AST (0-37 units/L)	ALT (0-40 units/L)	T. Bili (0.0-1.0 mg/dL)	PT (11-13.5)
1	2136	2321	7.2	11.3
2	1912	2267	7.4	11.9
3	2005	2536	7.2	12
4	1771	2517	8.7	11.8
5	1717	2583	9.5	11.5
6	1395	2250	8.6	11.5
7	1191	2147	9.2	11.4
8	1322	2217	10.5	11.6
9	1088	2039	10.2	10.9
10	777	1688	7.8	10.9
11	766	1655	7.1	10.9
12	644	1377	5.9	10.8
13	475	1131	4.5	10.5
14	413	985	4	10.5
15	343	868	4.1	10.5
16	306	772	4.1	10.5
17	248	666	3.4	10.5

## Discussion

This case of DILI in a patient undergoing TB treatment provides valuable insights into the clinical presentation, diagnosis, and management of DILI. The patient presented to the emergency department with markedly elevated liver enzymes, indicating potential liver dysfunction. Elevated levels of ALT and AST are clinically significant when they exceed three times the upper limit of the normal range, as mild elevations are common in various conditions. In this case, the enzyme levels were five times above the normal range, accompanied by a T.Bili of 7.2 mg/dL, suggesting acute hepatocellular injury [5]. A hepatitis panel that included hepatitis A, B, C, D, and E ruled out viral hepatitis.

DILI remains a diagnosis of exclusion, necessitating a comprehensive evaluation to rule out other causes of liver enzyme elevation. In addition to the hepatitis panel, an abdominal ultrasound was performed, revealing a contracted gallbladder but no other significant hepatobiliary, parenchymal, or vasculopathy abnormalities [6]. An abdominal CT scan was also unremarkable. The patient's history and clinical course raised suspicion for isoniazid-induced liver injury, given that anti-TB drugs like isoniazid are well-documented causes of idiosyncratic DILI [7]. Isoniazid, a first-line agent for TB, is effective but commonly associated with hepatotoxicity, often presenting weeks to months after treatment initiation [7]. Most cases are asymptomatic, with only alterations in liver biochemistry being noted, typically exceeding three times the normal upper limit for AST and ALT [7]. Clinical manifestations of isoniazid-induced liver injury, such as nausea, vomiting, abdominal pain, and jaundice, were observed in this patient, reinforcing the likelihood of DILI as the diagnosis.

The MELD score is a critical tool for estimating liver-related mortality, incorporating serum bilirubin, creatinine, and INR values [8]. Higher MELD scores correlate with increased mortality risk; this patient's admission MELD score of 18 indicated a moderate significant risk. In cases where the MELD score does not improve, it can be instrumental in prioritizing patients for liver transplantation. Regular MELD score calculations were recommended throughout the clinical course to assess prognosis and guide management decisions.

The cornerstone of DILI management is the immediate cessation of the offending agent. In this case, the patient was considered a slow acetylator of isoniazid (INH), which increased the risk of INH-associated toxicity [9]. Individualized dosing based on acetylator status and liver function may help mitigate toxicity, but discontinuation of the drug is often necessary when such options are unavailable. The clinical team opted to withdraw all anti-TB medications until the patient's liver enzymes stabilized. NAC, primarily used in acetaminophen overdose, has also demonstrated efficacy in non-acetaminophen DILI due to its antioxidant and anti-inflammatory properties [4]. Despite stopping INH, the patient's liver enzymes continued to rise, necessitating NAC therapy, which resulted in a gradual improvement in liver function tests. 

**Figure 2:**
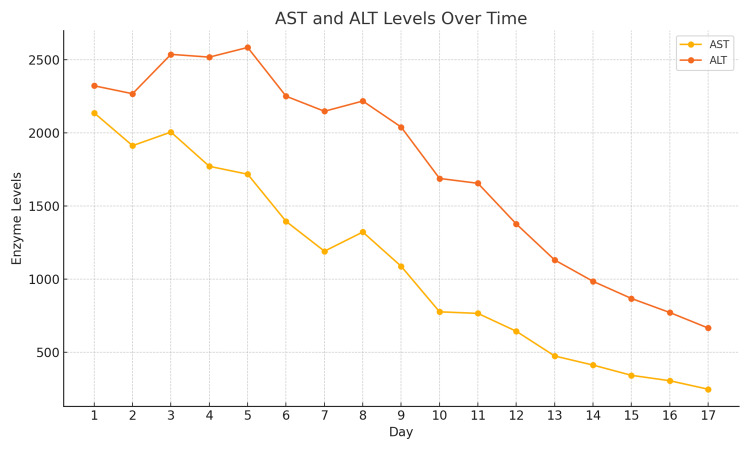
Aspartate aminotransferase (AST) and alanine aminotransferase (ALT) levels over 17 days. The trends indicate a gradual decline in both levels across the period, indicating recovery or improvement.

**Figure 3:**
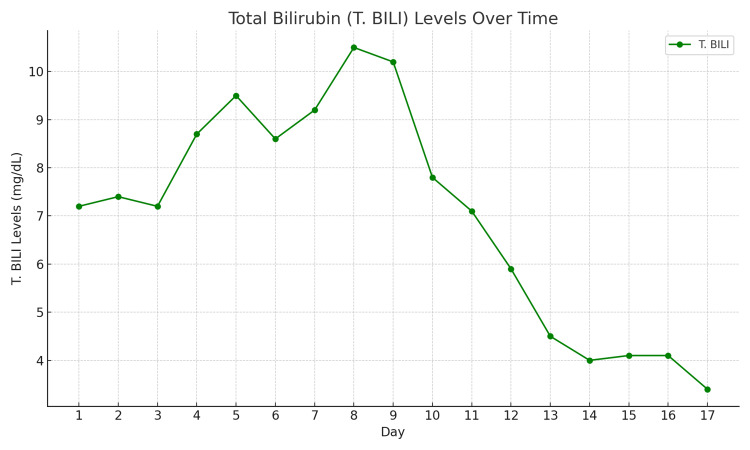
Total bilirubin (T.Bili) levels over the 17-day period. The levels increased initially, peaking around days eight and nine, before gradually declining.

**Figure 4:**
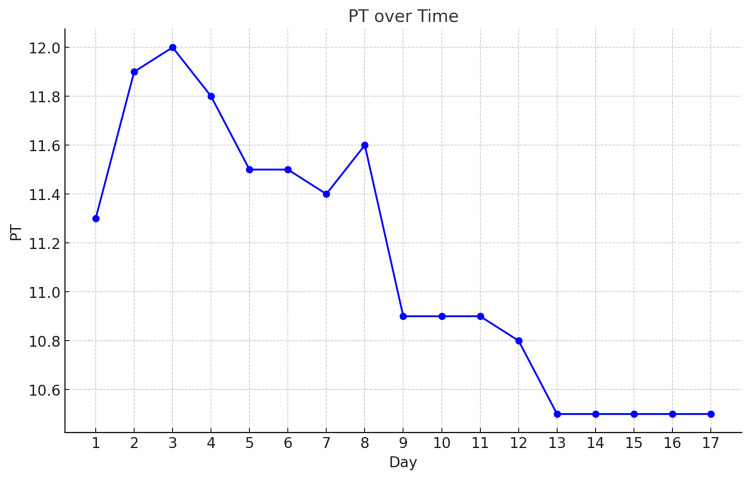
Prothrombin time (PT) values over 17 days. The data shows a peak on day three and a gradual decline from day nine onward, stabilizing at 10.5 from day 13 to day 17.

After the third dose of NAC, the patient showed a steady decline in liver enzyme levels, and the MELD score improved, suggesting effective treatment. This improvement reduced the need for liver transplantation. Reintroducing anti-TB medications without exacerbating liver dysfunction requires careful consideration to avoid drug resistance and assess the need for non-hepatotoxic second-line options (Figures 2-4).

NAC, a derivative of the amino acid L-cysteine, has been extensively studied for its diverse pharmacological properties. Initially used as a mucolytic agent and an antidote for acetaminophen overdose, NAC has gained attention for its potential therapeutic applications in various conditions due to its anti-inflammatory, antioxidant, and neuroprotective effects [10]. 

Although no specific guidelines have been established regarding the optimal timing for NAC administration, this case report suggests that early initiation of NAC may help reduce further liver injury caused by preformed toxins. In this report, liver enzyme levels remained persistently elevated during the first two weeks after the patient discontinued the offending agent. The subsequent rise in liver enzymes, 2-3 weeks post-discontinuation, can likely be attributed to ongoing liver injury due to the continued presence of preformed toxins. Notably, a rapid decline in liver enzyme levels was observed following the initiation of NAC, highlighting its potential benefit in mitigating further hepatic damage. This case underscores the possibility that early NAC intervention may play a role in limiting continued liver injury in such circumstances.

The primary mechanism of NAC involves replenishing intracellular glutathione levels, a critical antioxidant that protects cells against oxidative stress. NAC is a precursor to cysteine, necessary for glutathione synthesis [11]. By increasing glutathione levels, NAC helps neutralize reactive oxygen species (ROS), thereby reducing oxidative tissue damage. This antioxidant property supports its potential use in conditions characterized by oxidative stress, such as chronic obstructive pulmonary disease (COPD) and cardiovascular diseases. NAC's anti-inflammatory effects are primarily mediated through its ability to modulate the nuclear factor kappa B (NF-κB) pathway, which is central to inflammatory responses. By inhibiting NF-κB activation, NAC reduces the production of pro-inflammatory cytokines, as demonstrated in both in vitro and in vivo studies [10]. This mechanism underpins NAC's potential use in treating chronic inflammatory diseases like rheumatoid arthritis and inflammatory bowel disease (IBD) [12,13]. 

Although NAC is primarily used in managing acetaminophen overdose, it has shown promise in treating non-acetaminophen DILI due to its antioxidant and anti-inflammatory properties [4]. NAC's potential as a therapeutic agent extends to managing toxicities from drugs metabolized through acetylation, such as isoniazid. Isoniazid undergoes hepatic acetylation, a process that can produce toxic metabolites, especially in slow acetylators, leading to hepatotoxicity and neurotoxicity, including severe liver damage and peripheral neuropathy [14]. NAC may help in such cases by replenishing glutathione levels and detoxifying reactive metabolites, thereby reducing liver damage and neurotoxicity from isoniazid overdose [15].

Isoniazid (INH) is primarily metabolized in the liver through acetylation, a process catalyzed by the enzyme N-acetyltransferase 2 (NAT2) [15]. This metabolic pathway produces both active and toxic metabolites. The rate of isoniazid metabolism varies significantly among individuals due to genetic polymorphisms in NAT2. Some individuals are "fast acetylators," while others are "slow acetylators." Slow acetylators are at a higher risk of drug accumulation, which can lead to hepatotoxicity.

NAC may help mitigate isoniazid-induced liver injury due to its antioxidant properties. NAC acts as a precursor to glutathione, a critical antioxidant that neutralizes harmful metabolites produced during isoniazid metabolism. By replenishing glutathione levels, NAC may reduce oxidative stress and cellular damage in the liver.

Drugs such as hydralazine, an antihypertensive, and sulfonamides, a class of antibiotics, are metabolized through acetylation and can lead to toxicities, particularly in slow acetylators. Hydralazine is associated with adverse effects such as drug-induced lupus erythematosus (DILE), and NAC may help manage these toxicities due to its antioxidant properties and ability to modulate immune responses. However, more research is needed to confirm its efficacy [16]. Similarly, sulfonamides can cause hypersensitivity reactions and hepatotoxicity in slow acetylators, who accumulate higher levels of toxic metabolites. By enhancing glutathione synthesis and scavenging reactive species, NAC could reduce the severity of these toxicities, suggesting its potential as an adjunctive therapy despite limited direct evidence [17]. 

Although generally well-tolerated, NAC is not without side effects. Both oral and IV administrations can lead to gastrointestinal disturbances, such as nausea, vomiting, and diarrhea. In some cases, more serious adverse reactions, including anaphylactoid reactions, can occur, particularly with IV administration at high doses. The risk of these side effects is heightened with long-term use or in vulnerable populations, such as the elderly or those with compromised health [18]. 

NAC is a potent antioxidant that can cross the blood-brain barrier, helping reduce oxidative stress in the central nervous system. This property is central to its neuroprotective effects, which may benefit conditions like Parkinson’s disease, Alzheimer’s disease, and amyotrophic lateral sclerosis (ALS) by mitigating neuroinflammation and modulating neurotransmitter systems, including glutamatergic and dopaminergic pathways [19,20]. NAC's antioxidant properties also extend to psychiatric disorders, with clinical trials showing that it alleviates symptoms in schizophrenia, depression, and bipolar disorders by counteracting oxidative damage [20-22]. Furthermore, NAC may reduce cravings and prevent relapse in substance use disorders by restoring glutamate balance in the brain’s reward system [23,24].

## Conclusions

The clinical efficacy of NAC in many proposed applications remains controversial and under-researched. While robust evidence supports its use in acetaminophen overdose, the evidence for its effectiveness in treating neurodegenerative diseases, psychiatric disorders, and chronic inflammatory conditions is less conclusive. Although NAC is most commonly used for acetaminophen overdose, its potential protective effects against hepatotoxicity from other drugs, including isoniazid, are being explored, particularly in patients at risk for liver injury. However, clinical guidelines on its use, specifically for isoniazid toxicity, are limited, and further research is needed to establish optimal dosing and efficacy in this context.

In conclusion, this case report demonstrates the complexities of diagnosing and managing DILI, particularly in patients treated with hepatotoxic drugs like isoniazid. NAC shows promise as a therapeutic agent due to its antioxidant and detoxifying effects, helping manage toxicities associated with drugs that are metabolized through acetylation. While NAC has potential benefits for a range of conditions, including acetaminophen toxicity and some neurodegenerative and psychiatric disorders, its use must be carefully considered due to possible side effects and interactions. More robust research is needed to clarify NAC's effectiveness and safety across different clinical scenarios, ensuring its use is evidence-based and well-regulated.
